# Enhancing Obstetric Decision-Making With AI: A Systematic Review of AI Models for Predicting Mode of Delivery

**DOI:** 10.7759/cureus.83655

**Published:** 2025-05-07

**Authors:** Selma Mohammed Abdelgadir Elhabeeb, Sulafa Hassan Mahmoud Ali, Marwa Mohamed Ahmed Elkhidir Babikir, Fatima Siddig Abdalla Mohammed, Salma Hassan Mahmoud Ali, Nihal Ahmed Abd Elfrag Mohamed, Nihal Eltayeb Abdalla Elsheikh

**Affiliations:** 1 Obstetrics and Gynecology, Najran Armed Forces Hospital, Ministry of Defense Health Services, Najran, SAU; 2 Surgical Oncology, Prince Faisal Oncology Center, Buraydah, SAU; 3 Internal Medicine, Najran Armed Forces Hospital, Ministry of Defense Health Services, Najran, SAU; 4 Obstetrics and Gynecology, Armed Forces Hospital, Ministry of Defense Health Services, Wadi Al Dawasir, SAU

**Keywords:** artificial intelligence, machine learning models, mode of delivery prediction, obstetric decision-making, vaginal birth after cesarean (vbac)

## Abstract

Accurate prediction of the mode of delivery is critical for optimizing maternal and neonatal outcomes and reducing unnecessary cesarean sections. In recent years, AI has emerged as a promising tool for enhancing obstetric decision-making. This systematic review aimed to evaluate and synthesize existing evidence on AI models developed for predicting the mode of delivery, comparing their performance and clinical applicability across diverse settings. A comprehensive literature search was conducted to identify studies that developed and/or validated AI-based predictive models for mode of delivery outcomes, including vaginal birth after cesarean, emergent cesarean section during labor, and spontaneous vaginal delivery failure. Seventeen studies meeting inclusion criteria were analyzed, encompassing various AI models such as Random Forest, Gradient Boosting, XGBoost, CatBoost, support vector machines, neural networks, QLattice, and ensemble methods. Key study characteristics, input variables, model performance metrics, validation methods, and findings were systematically extracted and compared. The included studies, conducted across multiple countries and healthcare settings, demonstrated generally good to excellent predictive performance, with area under the curve values. Real-time intrapartum data significantly enhanced model accuracy in several studies. Ensemble models and advanced machine learning techniques outperformed traditional logistic regression in many cases, although simpler models remained competitive when interpretability was prioritized. Common predictive variables included maternal age, parity, BMI, previous cesarean, sonographic findings, and cervical examination data. Model transparency and external validation were highlighted as critical considerations for clinical translation. AI models show substantial potential for improving the prediction of the mode of delivery and supporting obstetric decision-making. Ensemble and real-time dynamic models demonstrated the highest performance. However, challenges remain regarding external validation, model interpretability, and integration into clinical practice.

## Introduction and background

The decision-making process surrounding the mode of delivery - whether vaginal or cesarean - is a cornerstone of obstetric care, with profound implications for maternal and neonatal outcomes [1]. Cesarean delivery rates have risen globally over recent decades, driven by complex factors such as maternal comorbidities, fetal distress, and evolving clinical guidelines [2]. However, this trend has sparked concerns about overmedicalization, unnecessary surgical risks, and long-term health consequences for both mothers and infants. Accurate prediction of the optimal delivery route remains a persistent challenge in obstetrics, as it hinges on synthesizing dynamic, multifactorial variables - ranging from maternal biometrics and obstetric history to intrapartum fetal monitoring - into a coherent clinical judgment [3]. Traditional approaches rely heavily on clinician experience and standardized protocols, yet variability in decision-making persists, underscoring the need for tools that enhance precision, reduce subjectivity, and align with evidence-based practices. In this context, AI has emerged as a transformative force, offering the potential to analyze vast, heterogeneous datasets and uncover patterns that may elude human practitioners, thereby refining predictive accuracy and supporting more individualized care [4].

The integration of AI into obstetric decision-making represents a paradigm shift, leveraging machine learning (ML) and deep learning (DL) techniques to model complex relationships between prenatal, intrapartum, and contextual factors [5]. Recent studies have explored AI’s capacity to predict delivery outcomes using diverse data sources, including electronic health records, ultrasound imaging, and continuous fetal monitoring tracings [6,7]. These models often incorporate variables such as maternal age, gestational diabetes, cervical dilation progression, and fetal weight estimates, aiming to stratify risks and inform timely interventions. For instance, neural networks trained on historical delivery data have demonstrated promise in identifying patients at high likelihood of cesarean delivery, enabling proactive resource allocation and patient counseling [8].

Despite the growing body of research on AI applications in obstetrics, the translation of these models into routine clinical practice remains limited. Concerns regarding the transparency, generalizability, ethical implications, and integration of AI tools into the clinical workflow persist. Moreover, the methodological heterogeneity across studies, including differences in dataset quality, model validation approaches, and outcome definitions, poses challenges in synthesizing the current evidence base. As such, a comprehensive and systematic examination of existing AI models designed for predicting the mode of delivery is crucial to identify promising approaches, highlight current limitations, and propose directions for future research and implementation.

This systematic review aims to critically evaluate and synthesize the available literature on AI-driven models for predicting the mode of delivery. By examining model types, input features, performance metrics, validation strategies, and clinical applicability, this review seeks to provide a clearer understanding of the current state of the field. In doing so, it aspires to contribute to the ongoing efforts to harness AI for safer, more equitable, and more personalized obstetric care, ultimately improving outcomes for mothers and their newborns.

## Review

Methodology

Study Design

This systematic review adhered to the Preferred Reporting Items for Systematic reviews and Meta-Analyses (PRISMA) guidelines [9] to ensure methodological rigor and transparency.

Eligibility Criteria

Studies were included if they (1) developed or validated AI or ML models to predict the mode of delivery (vaginal birth, cesarean section, or specific outcomes such as vaginal birth after cesarean (VBAC), emergent cesarean delivery, or induction success); (2) involved obstetric populations, including nulliparous or multiparous women, those with prior cesarean deliveries, or individuals undergoing labor induction; (3) were published in peer-reviewed journals or preprint servers; and (4) reported quantitative performance metrics. Exclusion criteria included non-English studies, animal research, editorials, conference abstracts without full-text data, and studies focused on non-obstetric populations or non-predictive AI applications (e.g., diagnostic imaging without delivery outcome prediction). No restrictions were placed on publication date or geographic setting.

Information Sources

A comprehensive search was conducted across four electronic databases: PubMed, Embase, Scopus, and Web of Science. Preprint servers (medRxiv and bioRxiv) and gray literature sources were also screened to mitigate publication bias. Additionally, reference lists of included studies and relevant systematic reviews were manually searched to identify additional eligible studies.

Search Strategy

The search strategy was developed in collaboration with a medical librarian and combined terms related to AI/ML (e.g., “artificial intelligence,” “machine learning,” “neural network,” and “random forest”) with obstetric delivery outcomes (e.g., “mode of delivery,” “cesarean section,” “vaginal birth,” “VBAC,” and “labor induction”). Boolean operators (AND/OR) and Medical Subject Headings were utilized. The detailed search strings for each database are presented in Table 1.

**Table 1:** Search strategies for different databases used in this study

Database	Search query
PubMed	("Artificial Intelligence"[Mesh] OR "Machine Learning"[Mesh] OR "Deep Learning"[Mesh] OR "artificial intelligence"[tiab] OR "machine learning"[tiab] OR "deep learning"[tiab] OR "neural networks"[tiab] OR "AI models"[tiab]) AND ("Obstetrics"[Mesh] OR "Pregnancy"[Mesh] OR "Labor, Obstetric"[Mesh] OR "mode of delivery"[tiab] OR "delivery prediction"[tiab] OR "cesarean section"[tiab] OR "vaginal delivery"[tiab] OR "birth mode"[tiab]) AND ("Decision Making"[Mesh] OR "decision-making"[tiab] OR "clinical decision"[tiab] OR "obstetric decision"[tiab] OR "prediction"[tiab])
Scopus	(TITLE-ABS-KEY("artificial intelligence" OR "machine learning" OR "deep learning" OR "neural networks" OR "AI models")) AND (TITLE-ABS-KEY("obstetrics" OR "pregnancy" OR "labor" OR "mode of delivery" OR "delivery prediction" OR "cesarean section" OR "vaginal delivery" OR "birth mode")) AND (TITLE-ABS-KEY("decision making" OR "clinical decision" OR "obstetric decision" OR "prediction"))
Embase	('artificial intelligence'/exp OR 'machine learning'/exp OR 'deep learning'/exp OR 'neural network'/exp OR 'artificial intelligence':ti,ab OR 'machine learning':ti,ab OR 'deep learning':ti,ab OR 'neural networks':ti,ab OR 'AI models':ti,ab) AND ('obstetrics'/exp OR 'pregnancy'/exp OR 'labor'/exp OR 'delivery prediction':ti,ab OR 'mode of delivery':ti,ab OR 'cesarean section':ti,ab OR 'vaginal delivery':ti,ab OR 'birth mode':ti,ab) AND ('decision making'/exp OR 'clinical decision':ti,ab OR 'obstetric decision':ti,ab OR 'prediction':ti,ab)
Web of Science	TS=("artificial intelligence" OR "machine learning" OR "deep learning" OR "neural networks" OR "AI models") AND TS=("obstetrics" OR "pregnancy" OR "labor" OR "mode of delivery" OR "delivery prediction" OR "cesarean section" OR "vaginal delivery" OR "birth mode") AND TS=("decision making" OR "clinical decision" OR "obstetric decision" OR "prediction")

Selection Process

The selection process involved two independent reviewers (SMAE and NEAE), who are experts in the field and, from the list of authors, screened titles and abstracts of all retrieved articles against the predefined eligibility criteria. Full texts of potentially eligible articles were then retrieved and assessed independently by the same reviewers. Any disagreements during the selection process were resolved through discussion, and a third reviewer (SHMA), who served as a tiebreaker, was consulted when consensus could not be reached. The selection process was documented using a PRISMA flow diagram, detailing the numbers of studies identified, screened, included, and excluded, along with reasons for exclusions at the full-text screening stage.

Data Collection Process

For the data collection process, a standardized data extraction form was developed and piloted on a small subset of studies. Two reviewers independently extracted data from the included studies, with discrepancies resolved through discussion or adjudication by a third reviewer. Data items extracted included study characteristics (e.g., author, year, country, and study design), population characteristics (e.g., sample size, maternal age, and gestational age), AI model characteristics (e.g., type of model, input features, and training and validation methods), outcome measures (e.g., accuracy, sensitivity, specificity, and area under the curve (AUC)), and any information regarding clinical implementation or validation.

Data Items

The primary data items of interest encompassed the type of AI model used, the features included in model development, dataset characteristics, model performance metrics, and validation approaches. Secondary data items included funding sources, potential conflicts of interest, and information on clinical feasibility or acceptability.

Risk of Bias Assessment

The risk of bias for included studies was evaluated using the Prediction model Risk Of Bias ASsessment Tool (PROBAST) [10], which systematically assesses four domains: participants (appropriateness of the study population), predictors (definition and measurement of input variables), outcome (definition and ascertainment of the predicted endpoint), and analysis (model development and validation methods). Two independent reviewers conducted the assessments, with disagreements resolved through consensus or consultation with a third reviewer. Studies were classified as having low, high, or unclear risk of bias in each domain, with the overall risk determined by the highest risk level across domains. Emphasis was placed on evaluating external validation practices, transparency in predictor selection, and handling of overfitting or missing data to ensure methodological rigor.

Synthesis Methods

Synthesis methods involved a narrative synthesis of findings from the included studies due to the anticipated heterogeneity in AI model types, outcome definitions, and evaluation methods. The results were grouped according to the type of AI model used (e.g., ML versus DL), the nature of input features (e.g., clinical, imaging, and electronic health records), and the setting in which the models were developed or validated. If sufficient homogeneity was identified among subsets of studies, a meta-analysis was considered, using a random-effects model to account for between-study variability. However, due to expected methodological diversity, the primary method of synthesis remained qualitative.

Results

Study Selection Process

**Figure 1:**
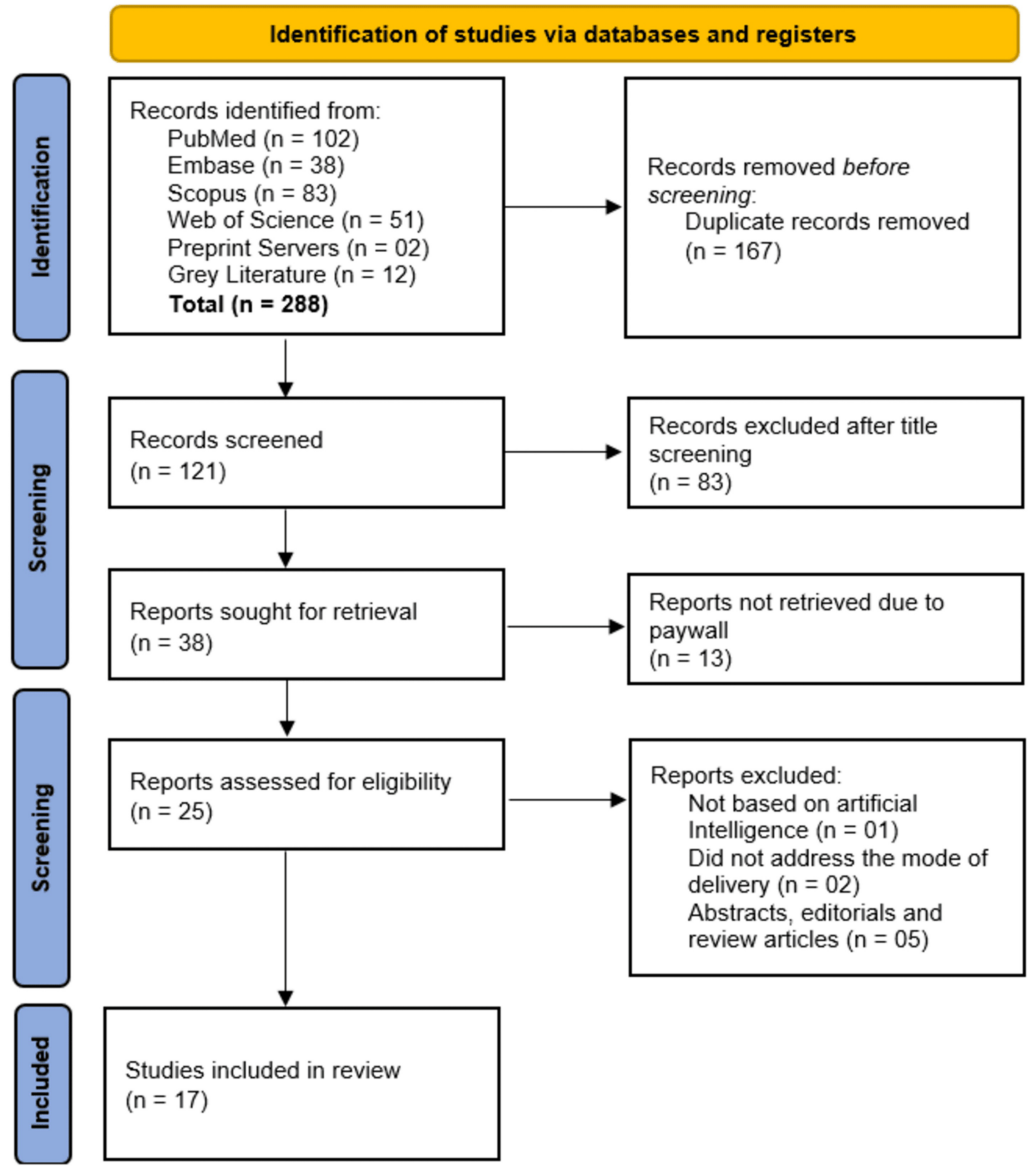
PRISMA flow diagram of study selection process PRISMA, Preferred Reporting Items for Systematic reviews and Meta-Analyses

The study selection process began with the identification of 288 records from multiple databases, including PubMed (n = 102), Embase (n = 38), Scopus (n = 83), Web of Science (n = 51), preprint servers (n = 2), and grey literature (n = 12). After removing 167 duplicate records, 121 studies underwent title screening, resulting in the exclusion of 83 irrelevant records. Of the remaining 38 reports sought for retrieval, 13 were unavailable due to paywalls, leaving 25 reports for eligibility assessment. Further exclusions were made for studies not based on AI (n = 1), those not addressing the mode of delivery (n = 2), and abstracts, editorials, or review articles (n = 5), culminating in 17 studies [11-27] included in the final review (Figure 1).

Characteristics of Included Studies

Seventeen studies [11-27] were included in this systematic review, conducted across various countries including Denmark, Sweden, Korea, Israel, the United States, China, Jordan, Taiwan, Ghana, Bangladesh, Spain, and Turkey. Most studies employed retrospective cohort designs, with sample sizes ranging from 40 to over 94,000 participants. The target populations varied, with most focusing on women undergoing trial of labor after cesarean (TOLAC), nulliparous term women, or women undergoing induction of labor. A wide range of AI models were used, including Random Forest, Gradient Boosting, XGBoost, CatBoost, support vector machines, neural networks, QLattice, and ensemble methods such as stacking and voting [11-13,17]. Some studies compared ML models against traditional logistic regression models, while others explored newer AutoML approaches [16] (Table 2).

**Table 2:** Characteristics of included studies evaluating AI models for predicting mode of delivery in obstetrics ANN, artificial neural network; AUC, area under the curve; AUROC, area under the receiver operating characteristic curve; CatBoost, categorical boosting; CS, cesarean section; DVI, dinoprostone vaginal insert; GLM, generalized linear model; KNN, K-nearest neighbors; LASSO, least absolute shrinkage and selection operator; LGBM, light gradient boosting machine; MFMU-C, Maternal-Fetal Medicine Units Network Cesarean prediction calculator; ML, machine learning; NB, Naive Bayes; ROC, receiver operating characteristic; SHAP, SHapley Additive exPlanations; SVD, spontaneous vaginal delivery; SVM, support vector machine; TOLAC, trial of labor after cesarean; VBAC, vaginal birth after cesarean; XGBoost, extreme gradient boosting

Author(s)	Year of publication	Country/setting	Study design	Sample size	Population characteristics	AI model/algorithm used	Input features/variables	Outcome predicted	Performance metrics	Validation method	Key findings
Thagaard et al. [11]	2024	Denmark	Cohort study	11,017	Women with prior cesareans giving birth between 2004 and 2016	QLattice, LASSO, Random Forest, Grobman’s logistic regression (baseline)	Antenatal model: epidural, breech presentation, mother's height, pre-pregnancy BMI, any vaginal birth, vaginal birth before cesarean; prelabor model: induction of labor, primary rupture of membranes, infant weight	Successful VBAC	AUC: antenatal model: QLattice 0.73, LASSO 0.75, Random Forest 0.74, Grobman 0.68; prelabor model: QLattice 0.77, LASSO 0.77, Random Forest 0.75, Grobman 0.70	Likely internal validation (specific method not mentioned in abstract)	QLattice performed comparably to other ML models with fewer variables and more explainability
Lindblad Wollmann et al. [12]	2021	Sweden (Stockholm-Gotland region)	Population-based cohort study	3,116 women	Women with one prior birth (cesarean) attempting trial of labor (no prior vaginal delivery)	Conditional inference tree, conditional random forest, Lasso binary regression	Not fully detailed in the abstract; likely maternal and obstetric characteristics	VBAC	AUROC (0.61-0.69), sensitivity (>91%), specificity (<22%), overall accuracy	Comparison with existing models (Grobman and Fagerberg models)	ML and classical models showed high sensitivity but low specificity; ML models did not outperform classical regression; most women with repeat cesareans were predicted to have a high chance (>60%) of VBAC
Wie et al. [13]	2022	Korea (nationwide multicenter dataset)	Multicenter retrospective cohort study	6,549 (development set), 1,391 (external validation set)	Term nulliparous women undergoing labor	Logistic regression, Random Forest, SVM, gradient boosting, XGBoost, LGBM, KNN, Voting, Stacking	Maternal age, height, pre-pregnancy weight, pregnancy-associated hypertension, gestational age, fetal sonographic findings	Emergent CS during active labor	C-statistic (up to 0.70), accuracy (up to 0.78), specificity (0.83), sensitivity (0.41)	External validation using a nationwide multicenter dataset	Logistic regression model performed best; ML models using clinical and sonographic data can predict emergent CS risk
D'Souza et al. [14]	2023	Data from two Phase III randomized controlled double-blind trials (NCT01127581 and NCT00308711)	Multivariate prediction model using ML	1,107	Singleton pregnancies, Bishop score <4, high-risk pregnancies undergoing induction with DVI	ML (type not specified)	Parity, gestational age (37-41 weeks), BMI, maternal age, maternal comorbidities, Bishop score	Vaginal birth following induction of labor	AUC = 0.73; parity alone approximately 50% correct predictions with approximately 10% false-negative rate	10-fold cross-validation	High vaginal birth rate (72%) despite low Bishop scores; parity critical predictor; judicious oxytocin use improves outcomes; ML models effective for predicting induction success
Guedalia et al. [15]	2020	Tertiary referral center (Israel)	Retrospective cohort study using electronic medical records	94,480 cases	Women undergoing trial of labor over a 12-year period	ML (specific algorithm not detailed)	Maternal and fetal parameters, cervical examination data, fetal heart rate data, additional labor data	Successful vaginal delivery	AUC ranging from 0.817 to 0.932, depending on model and timing	Not explicitly stated (likely internal validation using different stages of labor data)	Real-time labor data significantly improves the prediction of successful vaginal delivery; ML models can generate personalized risk scores to guide interventions
Wong et al. [16]	2024	Academic, tertiary care hospital (United States)	Retrospective cohort study	9,385 (Partometer cohort), 19,683 (control cohort)	Deliveries from 2013 to 2019 with at least two cervical examinations, divided by provider cesarean rates (<23.9% and others)	Supervised automated ML (stacked model)	Admission prediction model, cervical dilation, fetal station, ongoing intrapartum measures	Vaginal delivery probability at 4 hours after admission	Accuracy: 87.1%, ROC-AUC: 0.82, precision-recall AUC, calibration assessed	Internal comparison with the control model (different provider group)	Automated ML using intrapartum data improved prediction accuracy compared to traditional logistic regression models, showing potential for real-time clinical decision support to reduce maternal and neonatal morbidity
Xu et al. [17]	2024	China	Retrospective observational study	Not specified (needs full article for number)	Term nulliparous women, singleton pregnancy ≥37 weeks, no contraindications for vaginal delivery	Gaussian NB	Angle of progression, cervical length, subpubic arch angle, estimated fetal weight	SVD failure	AUC = 0.82 (training), AUC = 0.79 (validation); accuracy = 80.9%, sensitivity = 72.7%, specificity = 75.0%	Temporal validation set	The Gaussian NB model demonstrated good predictive performance for SVD failure before labor onset
Awawdeh et al. [18]	2023	Jordon	Retrospective study (implied from dataset usage)	659 pregnant women (327 with missing values)	Pregnant women with one previous lower segment cesarean section	Decision Tree, Random Forest, KNN, logistic regression	Individual characteristics of pregnancy (specific variables not detailed)	Mode of delivery (success of VBAC)	AUC: improved from 0.655 to 0.812 (KNN)	Not explicitly mentioned (likely internal validation)	Inputting missing data followed by feature selection improved prediction performance; KNN achieved the highest AUC (0.812)
Yang et al. [19]	2024	Northern Taiwan/regional hospital	Retrospective study	40 pregnant women (347 records)	Women with prior cesarean delivery considering VBAC	CatBoost (tree-based algorithm)	Gravidity, previous vaginal birth, and other medical record features (not all specified)	Successful VBAC prediction	Accuracy: 0.91 (95% CI: 0.86-0.94); AUC: 0.89 (95% CI: 0.86-0.93)	Not explicitly mentioned (likely internal validation; SHAP analysis for interpretation)	CatBoost outperformed logistic regression and other boosting models; identified gravidity and prior vaginal birth as key predictors; supported better shared decision-making
Meyer et al. [20]	2022	Tertiary academic medical center (Israel)	Retrospective Cohort	989	Singleton TOLAC deliveries	Random Forest, regularized regression (GLM), XGBoost	Previous vaginal delivery, maternal height, weight, prior arrest of dilation, prior arrest of descent, etc.	Success of TOLAC	AUC-PR: Random Forest (0.351 ± 0.028), XGBoost (0.350 ± 0.028), GLM (0.336 ± 0.024), MFMU-C (0.325 ± 0.067)	Nested cross-validation with 100 random splits (80% training, 20% testing)	ML models significantly outperformed MFMU-C; eight variables sufficient for accurate prediction; maternal height and prior arrest of descent were more important than weight and prior arrest of dilation
Macones et al. [21]	2001	Single institution (USA)	Retrospective cohort study	400 (100 failed, 300 successful trials of labor)	Women with prior cesarean delivery attempting trial of labor	Neural network (back-propagation algorithm) and multivariate predictive model (logistic regression)	History of substance abuse, prior VBAC, cervical dilatation at admission, need for labor augmentation	Success or failure of the trial of labor	Neural network: sensitivity 59%, specificity 44%; multivariate model: sensitivity 77%, specificity 65%, accuracy 69%	Train-test split (details not specified)	The multivariate predictive model outperformed the neural network in predicting trial of labor outcomes
Lipschuetz et al. [22]	2020	Tertiary referral center	Observational study (retrospective)	9,888	Singleton, term labor with previous cesarean delivery	Gradient boosting	Maternal and fetal features available at the first antenatal visit, and additional features available closer to delivery	Successful VBAC delivery	AUC: 0.745 (early features) and 0.793 (late features)	CIs (95% CI) for AUC	ML model predicts successful vaginal delivery after cesarean with high accuracy. Personal risk score and risk stratification help decision-making
Khan et al. [23]	2021	Bangladesh	Predictive modeling	Not mentioned	Not mentioned	XGBoost, AdaBoost, CatBoost	Amniotic liquid, medical indication, fetal intrapartum pH, number of previous cesareans, pre-induction	Necessity of Cesarean section	Accuracy: XGBoost (88.91%), AdaBoost (88.69%), CatBoost (87.66%)	Not mentioned	XGBoost showed the highest accuracy in predicting the necessity of cesarean section, with amniotic liquid, medical indication, and fetal pH being key features
Hu et al. [24]	2022	China	Cross-sectional	907	Primipara and multipara women	AdaBoost, logistic regression, NB, support vector machine	Risk factors for outcomes of induced labor (e.g., successful/failed induction)	Successful or failed induction of labor	Accuracy, recall, precision, F1 value, receiver operating characteristic curve	Not specified	Logistic regression performed best with an accuracy of 94.24% (primipara) and 96.55% (multipara) for successful induction and 65.00% (primipara) and 66.67% (multipara) for failed induction
De Ramón Fernández et al. [25]	2022	Spain (Murcia Region, Virgen of Arrixaca University Clinical Hospital)	Observational study	25,038 records	Women with singleton pregnancies, active labor, or planned induction of labor for medical reasons	Support vector machines, multilayer perceptron, Random Forest	48 clinical attributes	Mode of delivery: cesarean section, eutocic vaginal delivery, instrumental vaginal delivery	Accuracy ≥90% for classification between cesarean and vaginal deliveries; accuracy approximately 87% for classification between instrumental and eutocic deliveries	Not explicitly mentioned	AI algorithms can accurately predict the mode of delivery with performance above 90%, aiding decision-making in obstetrics.
Owusu-Adjei et al. [26]	2025	Ghana	Predictive modeling	Not mentioned	Maternal health features, real-time partographs	Gradient boosting, logistic regression, Random Forest	Gestational age, maternal cervix dilation, partograph data	Delivery type	Accuracy (gradient boosting 91%, logistic regression 93%, Random Forest 91%), balanced accuracy (gradient boosting 82.73%, logistic regression 84.62%, Random Forest 83.02%)	AUC, correlation statistic	Delivery type associated with gestational age and cervical dilatation. Logistic Regression had the highest accuracy
Beksac et al. [27]	2018	Turkey	Observational study	2,127 (1976), 3,548 (1986), 1,723 (1996)	Pregnant women undergoing delivery	ANN with backpropagation	Maternal age, gravida, parity, gestational age, labor induction type, fetal presentation, maternal disorders/risk factors	Vaginal delivery or cesarean section	Sensitivity: 60.9%, specificity: 97.5%, false positive rate: 2.5%, false negative rate: 39.1%, positive predictive value: 81.8%, negative predictive value: 93.1%	Not explicitly mentioned, but presumably based on historical data from 1976 to 1996	The “Adana System” can support delivery route prediction, offering high specificity and being useful for protecting against medicolegal issues

AI Models and Predictive Performance

The primary outcomes predicted were successful VBAC, emergency cesarean section during labor, spontaneous vaginal delivery failure, and general mode of delivery classification. Input features commonly included maternal demographics (age, height, and BMI), obstetric histories (parity and prior cesarean), sonographic measurements (estimated fetal weight and angle of progression), and intrapartum clinical parameters (cervical dilation and fetal station) [14,24,25].

Performance metrics varied across studies but generally showed good to excellent predictive ability. AUC values ranged from 0.61 to 0.932, with several studies reporting AUCs greater than 0.80 [15,16,19]. Logistic regression models remained competitive in some instances, particularly for induction success prediction [24]. However, ensemble models such as Random Forest, XGBoost, and CatBoost frequently achieved superior predictive performance. Real-time intrapartum data notably improved the accuracy of delivery mode predictions, as shown in studies by Guedalia et al. [15] and Wong et al. [16].

Model Interpretability and Clinical Implications

Several studies emphasized the importance of model interpretability for clinical use. Thagaard et al. [11] demonstrated that QLattice models could achieve comparable performance to more complex ML models with fewer predictors, enhancing transparency. Similarly, Yang et al. [19] used SHapley Additive exPlanations (SHAP) values in a CatBoost model to identify key predictors such as gravidity and previous vaginal birth for successful VBAC, supporting shared decision-making. Although ML models often outperformed traditional models in terms of AUC and accuracy, challenges such as low specificity in some settings [12] and the need for external validation remain important considerations. Overall, AI models showed substantial potential to enhance obstetric decision-making, particularly when combined with real-time clinical data.

Risk of Bias Assessment Results

Of the 17 studies assessed, 11 (65%) exhibited a high overall risk of bias, primarily due to limitations in the analysis and predictors domains. Common methodological shortcomings included reliance on internal validation alone [11,15], insufficient detail in predictor variable reporting [18], and inadequate handling of imbalanced outcomes [24]. Older studies [21] were further penalized for outdated modeling techniques and small sample sizes. In contrast, six studies (35%) demonstrated low overall risk, employing robust external validation [13], transparent predictor justification [16], and rigorous handling of confounders [14]. While most studies defined outcomes clearly (low risk in 88% of cases), the frequent absence of external validation and poor predictor transparency underscore critical gaps in generalizability and reproducibility, highlighting the need for standardized reporting and validation frameworks in AI-driven obstetric prediction models (Table 3).

**Table 3:** Risk of bias assessment of included studies using the PROBAST PROBAST, Prediction model Risk Of Bias ASsessment Tool

Study	Participants	Predictors	Outcome	Analysis	Overall risk of bias
Thagaard et al. [11]	Low	Unclear	Low	High	High
Lindblad Wollmann et al. [12]	Low	High	Low	High	High
Wie et al. [13]	Low	Low	High	Low	High
D'Souza et al. [14]	Low	Low	Low	Low	Low
Guedalia et al. [15]	Low	Unclear	Low	High	High
Wong et al. [16]	Low	Low	Low	Low	Low
Xu et al. [17]	Unclear	Unclear	Low	Unclear	High
Awawdeh et al. [18]	High	High	Low	Low	High
Yang et al. [19]	Low	Low	Low	Low	Low
Meyer et al. [20]	Low	Low	Low	Low	Low
Macones et al. [21]	High	High	Low	High	High
Lipschuetz et al. [22]	Low	Low	Low	Low	Low
Khan et al. [23]	Unclear	Unclear	Low	Unclear	High
Hu et al. [24]	Low	Low	High	High	High
De Ramón Fernández et al. [25]	Low	Low	Low	Low	Low
Owusu-Adjei et al. [26]	Unclear	Low	Low	High	High
Beksac et al. [27]	High	High	Low	High	High

Discussion

This systematic review synthesizes evidence from 17 studies conducted across diverse healthcare settings, demonstrating the growing interest in utilizing AI models to predict the mode of delivery and optimize obstetric decision-making. A major observation from the included studies is the broad range of AI algorithms employed, from traditional logistic regression to complex ML models such as Random Forest, Gradient Boosting, XGBoost, CatBoost, support vector machines, and neural networks, indicating a dynamic field of innovation that seeks to address persistent challenges in labor and delivery predictions. Notably, while various ML methods were applied, studies like those by Thagaard et al. [11] and Lindblad Wollmann et al. [12] demonstrated that explainability, model transparency, and clinical interpretability remain as crucial as predictive accuracy for the successful integration of AI into clinical practice.

One of the most significant findings across the included studies was the overall good to excellent predictive performance achieved by AI models. The AUC values reported ranged from moderate discrimination (0.61) to excellent (up to 0.932), highlighting that while AI models can substantially enhance prediction over chance, performance varies depending on the input features, the choice of model, and the population studied. Studies like Guedalia et al. [15] and Wong et al. [16] particularly stood out by demonstrating that real-time intrapartum data collection dramatically improved model performance, achieving AUCs greater than 0.80. This finding underscores the potential of AI to leverage dynamic, real-time clinical parameters to refine predictions, a concept that aligns with the broader trend in healthcare toward personalized, adaptive decision support tools.

Interestingly, while logistic regression models were often used as a comparator, several studies indicated that simpler models remained competitive, particularly when feature selection was optimal. Wie et al. [13] and Hu et al. [24] reported that logistic regression could achieve predictive performances comparable to more sophisticated models, especially when predicting outcomes like successful induction of labor. This observation is consistent with findings in other fields, where model simplicity often confers advantages in clinical acceptability, computational efficiency, and ease of interpretation, without substantial compromises in performance.

Model interpretability was another recurring theme across studies, with Thagaard et al. [11] introducing QLattice models that provided comparable accuracy to Random Forests but with fewer variables, promoting clinical explainability. Similarly, Yang et al. [19] utilized SHAP analysis within CatBoost models to offer intuitive insights into predictor importance, facilitating shared decision-making between patients and clinicians. These efforts to balance predictive accuracy with interpretability are crucial, as previous research emphasizes that clinicians are more likely to trust and adopt AI models that offer understandable reasoning pathways, as discussed by Rajkomar et al. [28] and Topol [29], who highlighted explainability as a linchpin for the ethical adoption of AI in medicine.

When examining the specific variables used for prediction, maternal age, BMI, parity, prior cesarean, fetal estimated weight, cervical dilation, and intrapartum sonographic measurements frequently emerged as critical predictors across multiple models [14,17,25]. This consistency suggests that, despite differences in settings and model architectures, certain clinical variables possess robust predictive value across populations. However, a few studies, such as those by D'Souza et al. [14] and De Ramón Fernández et al. [25], expanded the variable set to include maternal comorbidities and dynamic labor characteristics, indicating an evolving recognition of the multifactorial nature of labor outcomes.

Comparative analysis with external literature further supports these observations. For example, previous studies outside this review, such as those by Reddy et al. [30] and Deng et al. [31], have similarly identified maternal-fetal clinical features as powerful predictors of labor outcomes and have demonstrated that AI models trained on comprehensive clinical datasets outperform conventional risk calculators like the Maternal-Fetal Medicine Units VBAC calculator. These findings affirm the importance of comprehensive and quality input data in building robust predictive models, a concept widely acknowledged in data science literature, including the well-known principle of “garbage in, garbage out” in ML.

Despite these promising findings, challenges remain. One notable concern highlighted by Lindblad Wollmann et al. [12] is the issue of low specificity, even in models that achieved high sensitivity. High sensitivity is crucial for correctly identifying women likely to achieve successful vaginal birth, but low specificity increases the risk of false-positive predictions, potentially leading to inappropriate trial of labor recommendations. This limitation echoes broader criticisms of AI in healthcare, where models often perform well in development but encounter reduced effectiveness and unintended harms when implemented in real-world settings, as discussed by Cabitza et al. [32] regarding the “AI bias” phenomenon.

The necessity of robust external validation also emerged as a critical requirement. Although a few studies, such as Wie et al. [13] and Xu et al. [17], performed external validations using multicenter datasets, many others relied solely on internal validation or random splits of their own datasets. This methodological limitation raises concerns about overfitting and limits the generalizability of findings. Prior systematic reviews by Sendak et al. [33] and Nagendran et al. [34] have emphasized the importance of external validation to ensure that AI models maintain their performance across different patient populations, clinical workflows, and healthcare systems.

Another dimension of discussion relates to the context of AI application - real-time decision support versus pre-labor counseling. Studies like Guedalia et al. [15] and Wong et al. [16] effectively utilized real-time intrapartum data to update predictions continuously during labor, while others like Thagaard et al. [11] and Lipschuetz et al. [22] focused on antenatal or early labor assessments. Both approaches have clinical value; antenatal models assist with planning and counseling regarding TOLAC, whereas real-time models offer dynamic support to adjust strategies during active labor. Therefore, integrating both anticipatory and responsive AI tools may represent an ideal approach for optimizing obstetric outcomes, a strategy supported by contemporary frameworks for clinical AI implementation, such as the model proposed by Kelly et al. [35].

In comparing the findings from this systematic review with prior meta-analyses of VBAC prediction models, including those conducted by Grobman et al. [36] and Costantine et al. [37], it becomes evident that AI-driven models offer improvements in predictive performance. Classical models typically reported AUCs in the 0.70-0.75 range, while many AI models included in this review exceeded an AUC of 0.80, particularly when using real-time labor data [15,16,27]. This advance is not merely statistical; even modest gains in predictive discrimination can have substantial clinical implications in obstetrics, where delivery decisions profoundly impact maternal and neonatal outcomes.

Moreover, this review uncovered promising innovations such as the use of ensemble learning techniques and automated ML (AutoML). Wong et al. [16] demonstrated that AutoML, which automates the model selection and hyperparameter tuning process, achieved superior predictive performance compared to manually developed logistic regression models. These findings align with the growing evidence from the broader ML community that AutoML can democratize access to powerful predictive models, a trend noted by He et al. [38] in their systematic overview of AutoML applications in medicine.

The findings of this review also carry important implications for clinical practice and policy. The demonstrated ability of AI models to stratify patients according to predicted delivery outcomes suggests that AI could be used to support personalized labor management strategies, potentially reducing the rates of unnecessary cesarean sections, a goal that aligns with recommendations from the World Health Organization [39] advocating for the optimization of cesarean use. However, it must be emphasized that AI should not replace clinical judgment but rather augment it, a caution echoed by top clinical AI scholars such as Obermeyer and Emanuel [40], who warned against overreliance on algorithms without critical oversight.

Importantly, the review revealed that many studies prioritized model development over implementation research. Few studies explored how their models could be integrated into clinical workflows, assessed clinician acceptance, or evaluated impacts on patient outcomes. Future research must address these gaps by conducting prospective studies, including randomized controlled trials of AI-driven decision support tools, to rigorously evaluate their real-world effectiveness and safety. These steps are necessary to move beyond proof-of-concept to actual clinical impact, as emphasized by Steinhubl et al. [41] in their vision for digital medicine.

Equity considerations also warrant attention. Most studies in this review were conducted in high-income settings or tertiary referral centers, raising questions about the applicability of the models in low-resource environments, where labor and delivery practices, patient demographics, and healthcare infrastructure differ. The study by Owusu-Adjei et al. [26] in Ghana provides a positive example of AI application in a low-resource setting, but more efforts are needed to ensure that the benefits of AI in obstetric care are equitably distributed, an issue strongly underscored by Adamson and Smith [42] in their exploration of AI fairness in healthcare.

The ethical and medico-legal implications of using AI to predict delivery outcomes should not be underestimated. Predictive models that influence high-stakes decisions like the mode of delivery must meet the highest standards of accuracy, transparency, and fairness to avoid potential harms. Moreover, clear guidelines regarding liability in cases where AI-informed decisions contribute to adverse outcomes are essential. These concerns are increasingly recognized in the broader discourse on AI ethics in medicine, as discussed in the landmark report by the European Commission’s High-Level Expert Group on AI [43].

## Conclusions

AI models offer substantial promise for enhancing obstetric decision-making by accurately predicting the mode of delivery. Ensemble methods, real-time data utilization, and interpretable models show particularly strong potential to support personalized and dynamic management strategies during labor. However, challenges related to external validation, specificity, clinical integration, and equitable deployment must be addressed to realize this promise fully. Future work should focus not only on improving predictive performance but also on embedding AI solutions into the complex realities of clinical practice, ensuring that advances in technology translate into genuine improvements in maternal and neonatal health outcomes worldwide.
